# Locally Applied Slow-Release of Minocycline Microspheres in the Treatment of Peri-Implant Mucositis: An Experimental In Vivo Study

**DOI:** 10.3390/pharmaceutics12070668

**Published:** 2020-07-16

**Authors:** Sung-Wook Yoon, Myong-Ji Kim, Kyeong-Won Paeng, Kyeong Ae Yu, Chong-Kil Lee, Young Woo Song, Jae-Kook Cha, Mariano Sanz, Ui-Won Jung

**Affiliations:** 1 Department of Periodontology, Research Institute for Periodontal Regeneration, Yonsei University College of Dentistry, Seoul 03772, Korea; sungwook08@yuhs.ac (S.-W.Y.); mjikim212@yuhs.ac (M.-J.K.); kwp9310@yuhs.ac (K.-W.P.); tigger09@yuhs.ac (Y.W.S.); chajaekook@yuhs.ac (J.-K.C.); 2 College of Pharmacy, Chungbuk National University, Cheongju 28165, Korea; serom002@chungbuk.ac.kr (K.A.Y.); cklee@chungbuk.ac.kr (C.-K.L.); 3 Section of Graduate Periodontology, Faculty of Odontology, Complutense University of Madrid, 28040 Madrid, Spain; marianosanz@odon.ucm.es

**Keywords:** pharmacodynamic, drug sustainability, chitosan-alginate microspheres, peri-implant mucositis, local minocycline agent

## Abstract

Background: The objective of this is preclinical investigation was to evaluate the differential drug sustainability and pharmacodynamic properties of two local minocycline microsphere carriers: chitosan-coated alginate (CA) and poly(meth)acrylate-glycerin (PG). Methods: Four dental implants were placed unilaterally in the edentulous mandible of six beagle dogs. Each implant was randomly assigned to receive one of the following four treatments: (i) CA (CA-based minocycline), (ii) placebo (CA substrate without minocycline), (iii) PG (PG-based minocycline) and (iv) control (mechanical debridement only). After inducing peri-implant mucositis, the randomly assigned treatments were administered into the gingival sulcus twice at a 4-week interval using a plastic-tipped syringe. Drug sustainability and pharmacodynamic (clinical, radiographical and cell marker intensity) evaluations were performed after each administration. Results: The CA microspheres remained longer around the healing abutment compared to the PG microspheres at both administrations and a longer bacteriostatic effect was observed from CA (7.0 ± 5.7 days) compared to PG (1.2 ± 2.6 days). The efficacy of the applied therapies based on clinical, radiographical and histological analyses were comparable across all treatment groups. Conclusions: CA microspheres showed longer carrier and bacteriostatic effect sustainability when compared to PG microspheres, however, longer drug sustainability did not lead to improved treatment outcomes.

## 1. Introduction

Dental implants have demonstrated a high degree of predictability to rehabilitate absent teeth or tooth loss, with long-term (≥10 years) survival rates exceeding 94% [1]. Despite these survival rates, the prevalence of peri-implant diseases remains a major health problem. Peri-implant mucositis (mucositis) is defined by the presence of peri-implant tissue inflammation, identified by bleeding on probing (BOP) and by an increase in probing depth due to tissue swelling or a decrease in probing resistance, but without the evidence of peri-implant bone loss [2]. This condition occurs in about half of the population with dental implants [3,4,5], mostly in patients not adhering to regular supportive therapy. In fact, there is evidence that untreated mucositis frequently progresses to peri-implantitis [6], and therefore, its treatment is considered the most effective measure to prevent peri-implantitis [7]. The treatment of mucositis is based on effective infection control measures, since dental plaque accumulation on the implant/abutment surface is the critical etiological factor for its initiation and progression. In spite of some clinical studies reporting that professional and patient administered mechanical plaque control measures are effective in the control of mucositis, others have reported that improved clinical outcomes can only be achieved when combining mechanical therapies with chemical biofilm control [8]. Therefore, adjunctive therapies involving the local delivery of antimicrobial agents have been advocated [9]. Local delivery has the advantage of applying the antimicrobial agents at high concentrations at the targeted site with a relatively low systemic concentration [10]. However, the constant flow of the gingival crevicular fluid (GCF) and the cleansing activity of saliva may restrict their efficacy, which demands studies that evaluate the drug sustainability and pharmacodynamic properties of any proposed local antimicrobial agent for oral use [11].

Minocycline, a second generation tetracycline, due to its broad spectrum antimicrobial activity and long-term sustainability, has been formulated as minocycline-loaded microsphere agents and demonstrated improved clinical results when used as an adjunct to mechanical debridement therapy in the treatment of peri-implantitis [12]. Chitosan-coated alginate (CA) carriers have also been reported to have prolonged biodegradable sustainability for the controlled release of minocycline [13,14]. The use of this biopolymer has been advocated due to its biodegradability, improved wound healing, low toxicity and antimicrobial activity [15]. Other types of biodegradable polymers have included poly(meth)acrylate-glycerin (PG) microspheres with faster release of the contained chemical via diffusion control, thus contained minocycline is rapidly released to create a highly concentrated antimicrobial environment [16]. The aim of this experimental in vivo investigation was to evaluate drug sustainability and pharmacodynamic aspects of CA minocycline microspheres, when compared to PG microspheres, in an experimentally induced mucositis model in beagle dogs.

## 2. Materials and Methods

### 2.1. Experimental Animals, Housing and Husbandry

This preclinical in vivo study was approved by the International Animal Care and Use Committee, Yonsei Medical Research Center, Seoul, South Korea (Permission no.2017-31-0547) and followed the ARRIVE guidelines [17]. This experimental in vivo investigation was carried out in six male beagle dogs (12–15 months old) with a mean weight of 15 kg. All animals were sheltered under the supervision of professional veterinarians at Avison Biomedical Research Center of Yonsei University, Seoul, South Korea at a room temperature of 15–20 °C and humidity of ≥30%. The animals were quarantined for 2 weeks, and then were approved for use in the experiments after performing a careful health examination.

General anaesthesia was induced by inhalation anaesthesia with isoflurane followed by the intramuscular injection of xylazine (Rompun, BayerKorea, Seoul, South Korea) and the intravenous injection of atropine (Kwangmyung Pharmaceutical, Seoul, South Korea). Lidocaine HCl (2% with epinephrine 1:80,000; Kwangmyung Pharmaceutical) was injected at the surgical site for local anaesthesia. Intramuscular antibiotic injection (cefazolin sodium, Yuhan Pharmaceutical, Seoul, South Korea) was administered after the surgery.

### 2.2. Study Design

**Figure 1:**
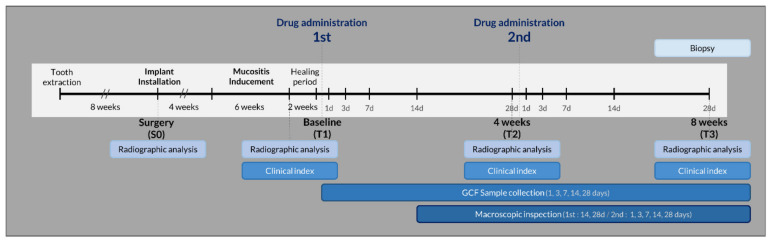
Study outline.

The study design is outlined in Figure 1.

### 2.3. Experimental Procedures

#### 2.3.1. Tooth Extraction and Implant Installation

**Figure 2:**
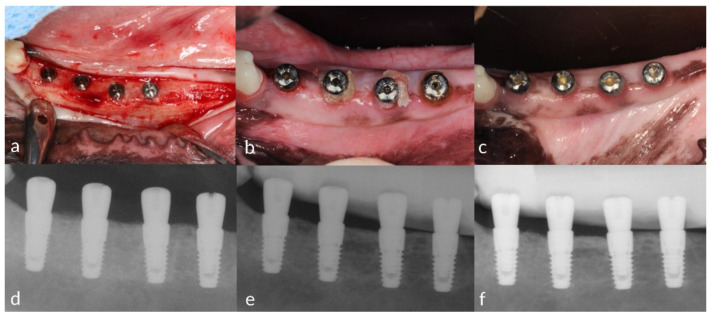
Clinical photographs and radiographs—Clinical photographs taken at (**a**) the surgery (S0), (**b**) the first drug administration (baseline, T1) and (**c**) 8 weeks after the baseline (T3). Peri-apical radiographs taken at (**d**) the surgery, (**e**) the baseline and (**f**) 8 weeks after the baseline.

The second to fourth premolars and the first molar were extracted unilaterally from the mandible of each dog. After an 8-week healing period, four implants were installed 7 mm apart at the level of the bone crest (Figure 2a). The implants were all of internal connection design with a sandblasted, large-grit, acid-etched surface and a size of 3.4 × 8 mm (Implantium; Dentium, Suwon, South Korea). Healing abutments (4.0 × 2.0 mm; Dentium) were then connected to the implant fixture and the wounds healed by primary closure. A 4-week healing period was applied during which plaque control was performed three or four times weekly using a toothbrush and chlorhexidine gluconate solution (hexamedine, Buk-wang Pharmaceutical, Seoul, South Korea).

#### 2.3.2. Experimental Peri-Implant Mucositis

Four weeks postoperatively, the oral hygiene measures were discontinued and animals were fed a soft diet for 6 weeks to induce plaque accumulation and develop mucositis [18]. New healing abutments (4.0 × 2.0 mm) were replaced after inducing mucositis.

#### 2.3.3. Antimicrobial Interventions

After professionally administered plaque control with an ultrasonic scaler, local delivery agents were placed into the gingival sulcus by means of a disposable injection syringe twice at a 4-week interval.

Each implant was randomly assigned to receive one of the following four treatments (Figure 2b):

1. 0.5 g of minocycline hydrochloride with CA microsphere carrier (CA; Minocline^®^, Dongkook Pharmaceutical, Seoul, South Korea),
2. CA microsphere carrier without the antimicrobial agent (placebo; prepared by the Dongkook Pharmaceutical company),
3. 0.5 g of minocycline hydrochloride with PG microsphere carrier (PG; Periocline^®^, Sunstar, Osaka, Japan),
4. mechanical debridement only (negative control).

#### 2.3.4. Drug Sustainability Evaluation

**Figure 3:**
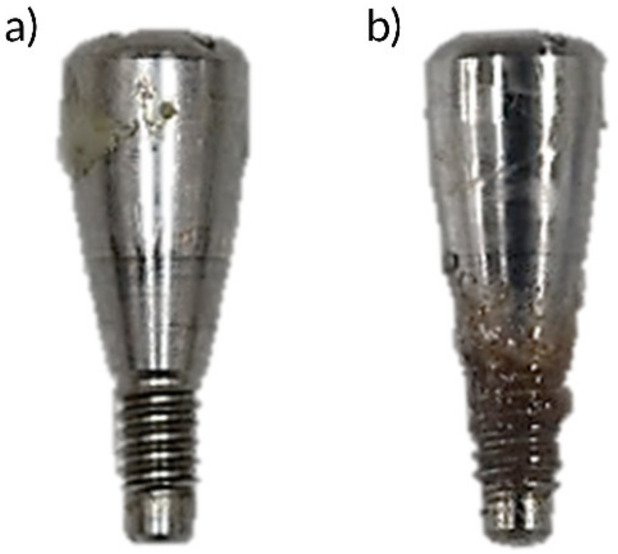
Macroscopic evaluation of carrier sustainability. (**a**) No residue and (**b**) residual agent visible at the subgingival part of healing abutment.

The sustainability of local drug agents was evaluated at macroscopical and microbiological level. To evaluate the sustainability of the local delivery agent carriers, healing abutments were retired from the implants of CA, placebo and PG groups, and circular inspection of the subgingival area proceeded macroscopically in plain sight for the presence of the residual antimicrobial agent (Figure 3) at 14 and 28 days after the first drug administration and at 1, 3, 7, 14 and 28 days after the second drug administration. The length of carrier sustainability was recorded as the time between the day of drug administration to the day when local delivery agents were no longer visible from the subgingival part of the healing abutments. The mean length of carrier sustainability was calculated as an average of all six implants from each of the CA, placebo and PG groups.

To assess the sustainability of the bacteriostatic effect, GCF and residual agent (if present) samples of the CA, PG and control groups were collected from the mesial, distal, buccal and lingual gingival sulcus around the healing abutments with a single stroke using a Gracey curette (Osung MND, Seoul, South Korea) at 1, 3, 7, 14 and 28 days after each drug administration. These samples were immediately placed in centrifuge plastic tubes (Eppendorf Tube^®^, Hamburg, Germany) containing 200 µL of distilled water and then stored in a deep freezer at −80 °C.

A single-blinded broth dilution assay was carried out by centrifuging the GCF sample solutions and serial dilution using phosphate-buffered saline (LPS solution, Daejeon, South Korea) [19]. A total amount of 100 µL of each diluted solution was added to a 96-well plate, and 5 µL of *Staphylococcus aureus* solution (2.5 × 10^7^ CFU) in LB broth (95 µL of 2× LB broth; BD Diagnostics, Sparks, MD, USA) was subsequently added to each well. After incubating the solutions for 24–48 h at 37 °C, bacterial cell growth was evaluated at 600 nm using a microplate reader (SpectraMax M2, Molecular Devices, San Jose, CA, USA).

#### 2.3.5. Clinical and Radiographical Outcomes

Clinical measurements were recorded in all the implants by a single examiner (M.J.K.) at the following time points: (a) baseline (T1), (b) 4 weeks after the first drug administration (T2) and (c) 4 weeks after the second drug administration (T3). BOP, plaque index (PLI) and gingival index (GI) [20] were measured to evaluate the severity of inflammation. Probing pocket depths (PPD) were measured using a periodontal probe with 1-mm marking (Qulix CP-15 UNC SE, Hu-Friedy, IL, USA).

Peri-apical radiographs of the implants were obtained using a customized film holder (XCP, Dentsply Rinn, Konstanz, Germany) with a portable X-ray device (DIOX-602, Digimed, Seoul, South Korea) (Figure 2d–f). The radiographs were obtained immediately after the fixture installation (Surgery, S0) and at 1, 2 and 6 weeks after plaque inducement to ensure that the marginal bone levels were maintained. Peri-apical radiographs were then obtained at T1, T2 and T3 to examine the bone level changes around the implant fixtures. The healing abutment–fixture junction (A/F) was marked as a reference point, and the average marginal bone level changes were recorded [21].

#### 2.3.6. Histological Preparation and Outcomes

The dogs were euthanized 30 days after the second administration with an overdose of sodium pentobarbital. The hemimandibles of the dogs were retrieved and the healing abutments were carefully removed. Tissue blocks prepared using a diamond saw were fixed in 10% buffered neutral formalin (Sigma Aldrich, Yong-in, South Korea) for 2 weeks and then were decalcified using 10% EDTA (Chelatocal, National Diagnostics, Atlanta, GA, USA). Before the blocks were fully decalcified, the fracture technique was performed to obtain two units from each block by slicing the centre of the buccal and lingual aspects parallel to the implant axis [22]. The decalcified blocks were then dehydrated with a graded series of ethanol concentrations. The units were embedded in paraffin and then sectioned at a thickness of 3 µm using an automated rotary microtome (Leica RM2255, Leica Biosystems, Nussloch, Germany). Each tissue section was then processed with immunohistochemistry (IHC) staining.

**Figure 4:**
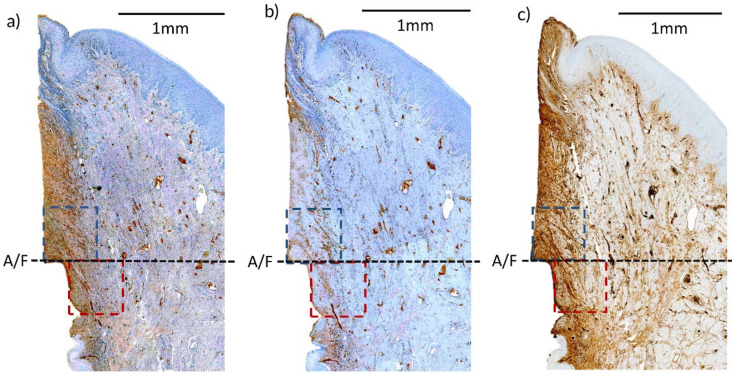
Immunohistochemical (IHC) stained histological slides of different types of primary antibody—(**a**) CD3, (**b**) CD20, (**c**) IgG antibody. A/F—abutment-fixture junction; upper (blue) and lower (maroon) region of interest (ROI) selected for cell marker intensity analysis.

The preparation for IHC included de-paraffinization with xylene prior to the staining. Sodium citrate buffer solutions (pH 6.0) were used for antigen retrieval. The sections were gently washed with Tris buffer saline (TBS) 0.025% Triton X-100 and then drained with tissue paper. Primary antibodies were diluted in TBS with 1% bovine serum albumin and were applied to the sections via a pipette. The sections were incubated overnight at 4 °C and then rinsed with TBS 0.025% Triton X-100 the following day. Horseradish peroxidase was used to conjugate with primary antibodies, and positive cells were detected using DAB (3,3’-diaminobenzidine) staining (Figure 4). The primary antibodies used in the IHC cell marker analysis were CD3 (ab828, Abcam, Cambridge, UK), CD20 (PA5-16701, Thermo Fisher scientific, Seoul, South Korea) and IgG (PAA544Ca01, Cloud-Clone corp., Katy, TX, USA). The specificity and dilution rates of the primary antibodies used in the IHC staining are listed in Appendix A, Table A1 [21].

Digital images of the specimens were obtained (Panoramic 250 Flash III, 3D, Case Viewer 2.0, 3D Histech, Budapest, Hungary) and the intensities of CD3, CD20 and IgG antibody staining were evaluated using the IHC profiler in Image J image-processing software [23]. Areas of 0.5 × 0.5 mm above and below the A/F were selected as regions of interest (ROIs) (Figure 4).

#### 2.3.7. Data Analysis

Statistical analysis was performed using standard software (SPSS version 25, IBM, Armonk, NY, USA). The mean values of the carrier sustainability length and bacteriostatic duration were calculated for each group and the mean values of measured variables were calculated for each implant and group in clinical, radiographical and histological evaluations. The null hypotheses of this study were as follows: 1. CA and PG will exhibit similar carrier degradability and bacteriostatic duration; 2. All treatment groups will present comparable treatment outcomes. Due to the small sample size, a non-parametric Kruskal—Wallis test was performed to compare the carrier sustainability length and bacteriostatic duration after each drug administration and to compare IHC cell marker intensity after sacrifice. If the results were significant (*p* < 0.05), a Mann—Whitney U test was performed as a post-hoc test with the significance criterion adjusted according to Bonferroni’s method (i.e., *p* < 0.0083). A Kruskal—Wallis test (*p* < 0.05) was also used to compare the clinical and radiographical outcomes at each time point (T1, T2 and T3) between the groups. To compare pre- and post-treatment differences within each group, a Wilcoxon signed-rank test (*p* < 0.05) was applied to assess intragroup treatment outcomes.

## 3. Results

The healing processes after tooth extraction and implant surgeries were uneventful at all surgical sites. Measures from all six dogs were included in the results.

### 3.1. Drug Sustainability Evaluation

#### 3.1.1. Carrier Sustainability

**Figure 5:**
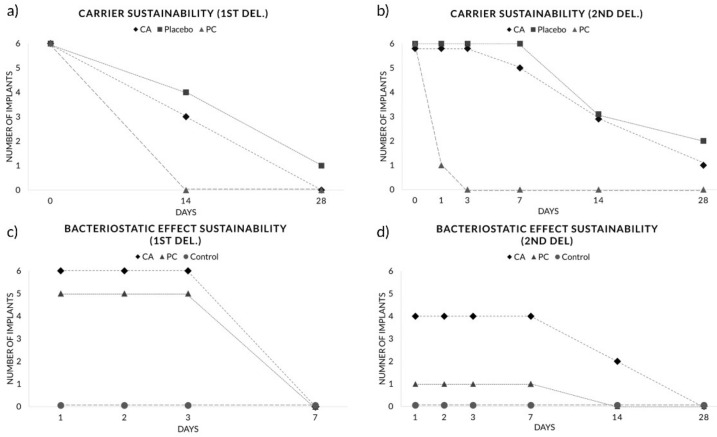
Evaluation of carrier and bacteriostatic effect sustainability—number of implants detected with residual agents at (**a**) the first administration and (**b**) the second administration. Number of implants exhibiting a bacteriostatic effect from broth dilution assay at (**c**) the first administration and (**d**) the second administration.

Macroscopic evaluation of the healing abutments revealed longer carrier sustainability with CA-based carriers (CA and placebo) compared to PG carrier. In the first administration, drug agent residues were detected from three implants (out of six) in CA (7.0 ± 7.0 days; *p* > 0.05, vs. PG) and four implants in the placebo (11.7 ± 9.6 days; *p* < 0.01, vs. PG) group 14 days after the administration, while no drug agent remnants were found from the PG group implants at day 14 (0.0 ± 0.0 days) (Figure 5a). In the second administration, drug agent residues were found in three implants from both CA (12.2 ± 8.1 days) and placebo (15.2 ± 9.4 days) groups at day 14, and two implants from the CA and one implant from the placebo group remained up to 28 days, while drug agent remnants were found in only one implant from the PG (0.2 ± 0.4 days; *p* = 0.002, vs. CA and placebo) group at day 1 and were all dissolved by day 3 (Figure 5b).

#### 3.1.2. Bacteriostatic Effect Sustainability

In the broth dilution assay, all six implants from the CA group presented the same bacteriostatic effect up to day 3 in the first drug administration. Five out of six implants from the PG group showed a bacteriostatic effect up to day 3 in the first drug administration (CA: 3.0 ± 0.0 days, PG: 2.5 ± 1.2 days; *p* > 0.05) (Figure 5c). In the second drug administration, two out of six implants from the CA group presented a bacteriostatic effect up to day 14 and two other implants were effective up to day 7 for CA (7.0 ± 5.7 days), while only one implant from the PG group lasted up to day 7 (1.2 ± 2.6 days, *p* = 0.132) (Figure 5d).

### 3.2. Clinical Findings

**Figure 6:**
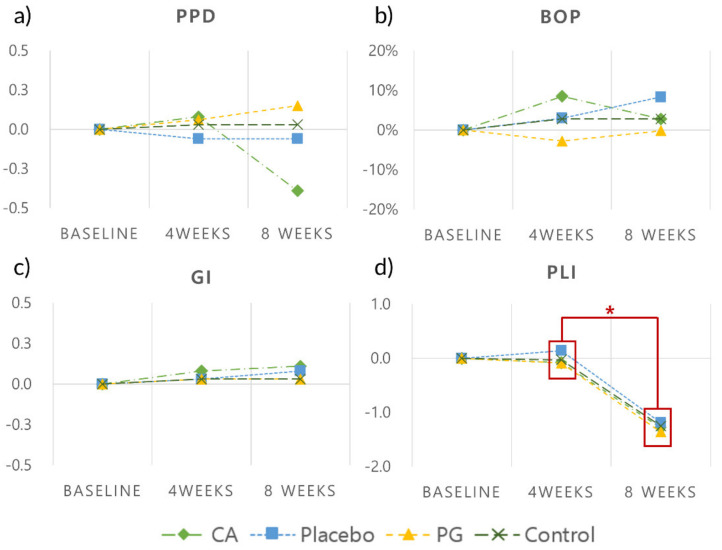
Interval changes of clinical parameters. (**a**) Probing pocket depth (mm); (**b**) bleeding on probing (%); (**c**) gingival index; (**d**) plaque index. * Statistical difference within each group (CA: *p* = 0.026, placebo: *p* = 0.027, PG: *p* = 0.028, control: *p* = 0.027).

Clinical parameters (mean PPD, BOP, GI and PLI values) at different time points are depicted in Figure 6 and Table 1. None of the clinical parameters differed significantly between the groups at T1, T2 and T3. Most of the interval changes were minimal, but all groups showed significantly reduced PLI between the first and second drug administration (*p =* 0.026, 0.027, 0.028 and 0.027 in the CA, placebo, PG and control groups, respectively). Furthermore, mean PPD was reduced in the CA group (−0.39 ± 0.49 mm), while other groups showed slight interval changes (−0.11 ± 0.18 mm (placebo) to 0.09 ± 0.40 mm (PG)) after the second drug administration.

### 3.3. Radiographical Findings

**Figure 7:**
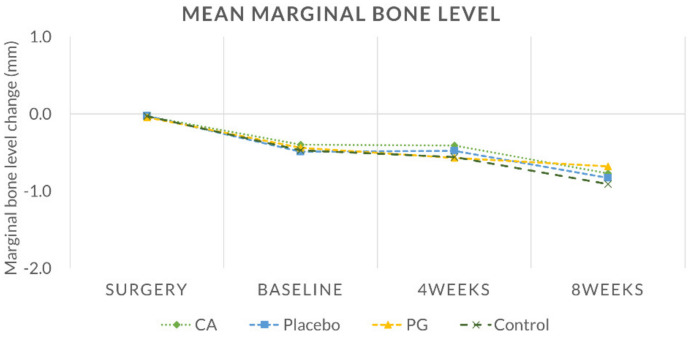
Mean marginal bone level change (mm).

Radiographical measurements from implant sites taken at S0, T1, T2 and T3 are presented in Figure 7. Mean bone level changes between S0 and T1 were −0.38 ± 0.26, −0.47 ± 0.30, −0.39 ± 0.38 and −0.44 ± 0.30 mm in the CA, placebo, PG and control groups, respectively, with no significant differences between any of the groups (*p* > 0.05). Negligible bone loss was observed in all treatment groups between T1 and T3 (−0.37 ± 0.26, −0.34 ± 0.23, −0.25 ± 0.23 and −0.44 ± 0.29 mm in the CA, placebo, PG and control groups, respectively (*p* > 0.05) (Appendix A, Table A2).

### 3.4. IHC Cell Marker Analysis

The cell marker intensities in the upper ROI, lower ROI and total mean (upper and lower ROI combined) are summarized in Table 2. The IHC cell marker intensities from all three antibodies (CD3, CD20 and IgG) were comparable between the groups. Moreover, there were no significant differences between the upper and lower ROIs within each group.

## 4. Discussion

This study evaluated drug sustainability and pharmacodynamic parameters when using two different carriers for local minocycline delivery in an experimentally induced mucositis environment. More implants from CA microspheres presented prolonged sustainability of the carrier (CA and placebo) and bacteriostatic effect (CA) compared to PG microspheres. Clinical, radiographical and IHC cell marker analysis presented comparable results between the groups.

The controlled release rate of minocycline in CA microspheres is primarily attributable to the stability of chitosan in aqueous solution. In CA microspheres, chitosan is coated around alginate microspheres via ionic coacervation using a cross-linking agent such as calcium chloride [13]. Chitosan is degraded inside the body by lysozyme, and so CA microspheres will dissolve and degrade in proportion to the amount of lysozyme present in the surrounding environment [24]. From a previous study which evaluated the biodegradability of a minocycline-loaded CA microsphere in relation to the concentration of chitosan in microsphere fabrication, a slower release rate of minocycline was observed [13]. Furthermore, this previous study reported a 50% microsphere weight decrease in two weeks in an in vitro setting of GCF with HEPES buffer and lysozyme (20 µg/mL) and suggested that a CA microsphere can maintain drug concentration for a week [13,25]. Among 12 CA microsphere-applied implants (CA and placebo), the local antibiotic agent remained for up to 14 days in 7 and 6 implants in the first and second trials, respectively, and for up to 28 days in 1 and 3 implants. With their slowly degradable property, a longer bacteriostatic effect was also observed in the broth dilution assay in the second administration (up to 14 days post-delivery) when compared to PG microspheres. A previous clinical trial which evaluated the effects of CA minocycline microspheres observed significant reduction in both aerobic and anaerobic bacterial counts even after 6 weeks and that BOP (%) and mean PPD were significantly reduced compared to the conventional supragingival scaling group [14].

On the other hand, PG carrier controls the drug release rate via diffusion. The microsphere is hydrophilic and comprises poly(meth)acrylate, glycerin and hydroxyethyl cellulose [26]. Therefore, when microspheres are locally injected into the gingival sulcus, they will instantaneously dissolve to release minocycline. A previous study of a locally injected PG minocycline microsphere reported that the minocycline concentration is immediately increased to 1300 µg/mL and then decreased to 90 µg/mL within 7 h after being delivered [16]. Another in vivo study which evaluated the drug retention rate of minocycline-loaded methoxy-poly(ethylene glycol) poly(lactic acid) nanoparticles with a PG minocycline microsphere determined the concentration of minocycline in GCF using high-performance liquid chromatography [27]. The study revealed that the minocycline concentration of the PG carrier fell under 3 µg/mL after 2 days post-delivery and the action of the drug (>1 µg/mL) lasted up to 8 days. Comparable to the present study, most locally injected agents were dissolved and the residual agent was detected in only one implant at day 1 from the PG group, and the bacteriostatic effect lasted up to day 7 during the second trial. However, care is needed before assuming that one agent is superior to the other, because the cumulative amount of minocycline released in the two types of microspheres may be equivalent since both the CA and PG groups contained 10 mg of minocycline hydrochloride. From the clinical results, the CA group showed a slight reduction of the mean PPD (0.4 mm) after the second administration, while the PG group showed a minimal increase (0.1 mm) over the same interval (Table 1). There was also a slight reduction of the mean BOP relative to the CA group (5.6%) after the second administration, while the PG group presented a slight increase (2.7%). However, this apparent difference between these two groups was not statistically significant.

In addition to the slow degradation of chitosan, CA microspheres also have the advantages of the anti-inflammatory effect of chitosan and the bio-adhesion ability of alginate to mucosa [28,29]. When minocycline-containing alginate microspheres are released to the gingival sulcus, alginate will adhere to the mucosa so as to prevent it from washing out due to the GCF and salivary flow. Furthermore, even when minocycline is completely released into the surrounding environment, chitosan can act as an anti-inflammatory material itself. Thaya et al. [29] reported antibacterial and antibiofilm activities of CA microspheres against Gram-positive and -negative species in in vitro tests. These observations indicate that CA microspheres are a highly effective carrier for the local delivery of antibacterial agents.

An immune response begins with cell-mediated immunity involving phagocytes and T-cells, and then initiates humoral immunity as helper T-cells stimulate B cells to differentiate to plasma and memory cells after undergoing mitosis. This means that B cells and plasma cells are mostly involved in the latter inflammatory stage. Concurrently, most (about 60%) of the inflammatory cells in periodontal disease are largely comprised of B cells and plasma cells [30]. The IHC cell marker analysis examined three different types of antibodies: CD3, CD20 and IgG. In the IgG cell marker analysis using an antibody specific to B cells and plasma cells, the CA group presented the lower tendency of cell marker intensity in both the upper and lower ROIs of the A/F among all other test groups. In the histological slide samples, infiltrated connective tissue (ICT), an area of which inflammatory cells are distributed, is present throughout the coronal and apical portion of the pocket along the implant fixture (Figure 4). Carcuac et. al. [21] reported that histological evaluation of ICT in experimentally induced peri-implantitis revealed larger ICT and longer ulcerated pocket epithelium compared to experimentally induced periodontitis due to lack of ability to encapsulate the lesion. A closer examination of the histological samples of this current study also revealed that pocket epitheliums were stretched to the bone crest with no epithelial barrier in the most apical part of the ICT.

The efficacy of an adjunctive local antibacterial therapy in periodontal or peri-implant disease is still controversial. A review article reported that the BOP tendency and probing depths can be reduced compared to those in non-surgical therapy by applying adjunctive local antibacterial delivery [31]. Other controlled clinical studies reported that the use of adjunctive local minocycline delivery after non-surgical therapy showed improved BOP, PPD and bacterial counts after 12 months of treatment [32,33]. However, in an in vivo study which evaluated the antiseptic effects of chlorhexidine in experimentally induced mucositis in nine cynomolgus monkeys, comparable mean PLI and GI scores and mean PPD reductions were observed between the mechanical treatment group and the adjunctively treated chlorohexidine group [34]. While significant clinical improvements were observed from the treatment groups when compared to the no treatment group, mechanical treatment alone was sufficient to achieve clinical resolutions of mucositis lesions. The clinical results from this study also demonstrated that the treatment outcome of mechanical debridement alone is comparable.

A recent randomized placebo-controlled clinical trial emphasized the importance of the repeated administration of local antibiotic agents after treatment [12]. In this clinical study, a local minocycline agent was delivered into the gingival sulcus of the patients with peri-implantitis at the surgical treatment, stitch-out, 1 month and 3 months post-operation. Improved clinical and radiographical results were observed in the minocycline ointment-treated group when compared to the placebo ointment-treated group. The present study also repeatedly injected the local antibiotic agent at a 1 month interval. Although a reduced mean PPD was observed from the CA group after the second administration, no statistical differences were observed in clinical and radiographical measures between the groups. The present authors carefully interpreted that the effect of a single delivery at a 1 month interval may have been an overly distant period to properly evaluate the effect of the repeated application of a local antibiotic agent since healing in dogs is believed to occur two to three times faster than in humans [35].

The discrepant treatment results from this in vivo study with the studies of those which presented an improved outcome when using adjunctive local antibacterial therapy may have been associated with some of the limitations of this study. The present study investigated the sustainability of the agents. Therefore, care was taken to ensure that the sulcular environment did not interfere with oral hygiene care after each delivery. For this reason, only the supragingival parts of the healing abutments were cleansed using a hydrogen peroxide-soaked gauze during the investigation period. Since oral hygiene control is known to be a critical factor in relieving inflammation, the authors carefully speculate that the effects of the restricted oral hygiene care may have surpassed the effects of local minocycline. Furthermore, the treatment responses varied between the included animals. The author evaluations of the periodontal status of the studied animals revealed that two dogs presented less-favourable soft-tissue healing patterns, showing more gingival swelling and remaining redness than the others. Due to the small size of the sample, such variations among individuals produced relatively large standard deviations that influenced the statistical power of the study.

This study evaluated the drug sustainability and pharmacodynamic parameters of local minocycline with different constituents on a monthly basis for two months. Following the ARRIVE guidelines, the authors only evaluated short-term clinical and radiographical outcomes. Moreover, histological data were collected only after a sacrifice, and so interval changes between before and after the treatment were not determined. Thus, further studies are needed to evaluate the long-term effects with a sufficient delivery interval and microbiological evaluations that can present interval changes.

## 5. Conclusions

Within the limitations of this study, drug sustainability evaluations from this study presented prolonged carrier sustainability and a bacteriostatic effect of CA minocycline-containing microspheres when compared to diffusion-controlled PG minocycline microspheres. However, prolonged sustainability of minocycline did not lead to improved treatment results.

## Figures and Tables

**Table 1:** Clinical parameters (mean ± S.D.) measured at different time points.

Clinical Parameter	Group	Baseline (T1)	4 Weeks (T2)	8 Weeks (T3)
Mean PPD (mm)	CA	2.56 ± 0.46	2.64 ± 0.49	2.25 ± 0.27
	Placebo	2.39 ± 0.31	2.44 ± 0.54	2.33 ± 0.36
	PG	2.41 ± 0.22	2.47 ± 0.20	2.56 ± 0.60
	Control	2.47 ± 0.30	2.50 ± 0.17	2.50 ± 0.44
*p*-value		0.975	0.847	0.585
Mean GI	CA	1.44 ± 0.23	1.53 ± 0.11	1.56 ± 0.33
	Placebo	1.56 ± 0.25	1.58 ± 0.13	1.64 ± 0.15
	PG	1.44 ± 0.21	1.44 ± 0.18	1.47 ± 0.31
	Control	1.42 ± 0.08	1.44 ± 0.08	1.44 ± 0.23
*p*-value		0.680	0.267	0.501
Mean BOP (%)	CA	44.3 ± 22.9	52.8 ± 11.7	47.2 ± 26.2
	Placebo	55.5 ± 24.7	58.5 ± 13.0	63.8 ± 14.8
	PG	47.3 ± 22.5	44.5 ± 18.5	47.2 ± 31.1
	Control	41.5 ± 8.5	44.3 ± 8.0	44.3 ± 22.9
*p*-value		0.646	0.267	0.449
Mean PLI	CA	2.75 ± 0.23	2.67 ± 0.22	1.47 ± 0.40 ^‡^
	Placebo	2.64 ± 0.22	2.78 ± 0.18	1.45 ± 0.41 ^‡^
	PG	2.78 ± 0.25	2.69 ± 0.32	1.42 ± 0.37 ^‡^
	Control	2.72 ± 0.25	2.70 ± 0.20	1.47 ± 0.37 ^‡^
*p*-value		0.760	0.811	0.972

*p*-value: group comparison (Kruskal-Wallis test) at each time point; ^‡^ significantly different from T2 within each group (Wilcoxon signed-rank test, *p* < 0.05). PPD—Probing pocket depth, GI—Gingival index, BOP—Bleeding on probing, PLI—Plaque index.

**Table 2:** IHC cell marker analysis.

		CA	Placebo	PG	Control	*p*-Value
CD3 (%)	Upper	8.60 ± 4.27	8.66 ± 4.05	8.70 ± 5.35	6.60 ± 4.41	0.615
	Lower	8.30 ± 3.90	7.49 ± 4.50	7.59 ± 4.57	5.94 ± 5.43	0.340
	Total mean	8.60 ± 4.27	8.02 ± 4.34	8.26 ± 5.08	6.31 ± 4.90	
CD20 (%)	Upper	6.35 ± 3.38	6.70 ± 3.89	4.34 ± 2.51	6.26 ± 3.54	0.572
	Lower	6.76 ± 7.71	7.57 ± 5.16	5.46 ± 2.20	7.36 ± 4.01	0.539
	Total mean	6.55 ± 5.86	7.10 ± 4.54	4.90 ± 2.43	6.84 ± 3.83	
IgG (%)	Upper	6.33 ± 3.52	15.18 ± 10.95	8.34 ± 7.16	11.20 ± 9.33	0.226
	Lower	6.14 ± 5.49	10.16 ± 7.95	8.82 ± 6.56	6.14 ± 3.73	0.369
	Total mean	6.23 ± 4.61	12.78 ± 9.95	8.61 ± 6.83	8.67 ± 7.54	

The numbers represent mean percentage of cell marker intensity distribution of upper ROI, lower ROI and mean score of the two ROIs combined (total mean). They are summated scores of high positive and positive values from IHC profiler, Image J.

## Appendix A

**Table A1:** Types of monoclonal antibodies used for IHC staining.

Antibody	Specificity	Dilutions	Source
CD3	T cells	1:200	Abcam
CD20	B cells	1:800	Thermo Fisher scientific
IgG	Plasma/B Cells	1:800	Cloud-Clone corp.

**Table A2:** Mean radiographical interval changes of marginal bone level (mm).

	ΔS0–T1	ΔT1–T2	ΔT2–T3	ΔT1–T3
CA	−0.38 ± 0.26	−0.01 ± 0.20	−0.36 ± 0.29	−0.37 ± 0.26
Placebo	−0.47 ± 0.30	0.01 ± 0.02	−0.35 ± 0.24	−0.34 ± 0.23
PG	−0.39 ± 0.38	−0.14 ± 0.17	−0.11 ± 0.15	−0.25 ± 0.23
Control	−0.44 ± 0.30	−0.09 ± 0.18	−0.35 ± 0.26	−0.44 ± 0.29
*p*-value	0.813	0.439	0.181	0.590
